# Effects of the Digital Economy on Carbon Emissions: Evidence from China

**DOI:** 10.3390/ijerph19159450

**Published:** 2022-08-02

**Authors:** Zhichuan Zhu, Bo Liu, Zhuoxi Yu, Jianhong Cao

**Affiliations:** 1 School of Economics, Liaoning University, Shenyang 110136, China; liubolnu@126.com (B.L.); yzx8170561@163.com (Z.Y.); 2 School of Mathematics and Statistics, Liaoning University, Shenyang 110031, China; 3 School of Business and Economics, Universit Putra Malaysia, Serdang 43400, Malaysia; xiatiandeguoguo@gmail.com

**Keywords:** digital economy, carbon emissions, sustainable development, spatial spillover, China

## Abstract

In order to reduce carbon emissions for sustainable development, we analyzed the impact of China’s digital economy development on carbon emissions. Based on the panel data of 30 Chinese provinces from 2009 to 2019, we measured the level of development of China’s digital economy using the entropy method. The relationship between the digital economy and carbon emissions was analyzed from multiple perspectives with the help of the fixed-effects model, the mediated-effects model and the spatial econometric model. The results indicate that the digital economy plays a significant inhibitory role in carbon emissions. In addition, the digital economy inhibits carbon emissions through the innovation effect and the industrial structure upgrading effect. Moreover, the digital economy exhibits a significant spatial spillover effect in dampening carbon emissions. Finally, there is regional heterogeneity in the direct and spatial spillover effect. The findings provide a basis for the digital economy to contribute to carbon emissions reduction and provide relevant policy references for achieving carbon neutrality and sustainable development.

## 1. Introduction

With the steady progress of reform and the opening-up process in China, the country’s economic development has been greatly enhanced, creating an “economic growth miracle” rarely experienced in the world [1]. However, this factor-driven extensive growth has also led to a large consumption of energy and resources in China, resulting in high carbon emissions [2]. According to the BP Statistical Review of World Energy, China has been the world’s fastest-growing energy consumer for 19 consecutive years [3] and accounts for 29% of the world’s total carbon emissions [4]. Although carbon emissions show a certain positive association with economic growth [5], the large amount of carbon emissions will not only impact the climate [6], but also human health. Previous studies have shown that carbon dioxide can cause harm to human health through inhalation and skin contact [7,8]. Large amounts of carbon emissions constrain long-term human development by affecting the climate and thus, indirectly, human health [9,10]. Such effects include, unavoidable injuries and deaths caused by extreme weather such as floods, storms and hurricanes; respiratory diseases, asthma, and allergies; sunburn and skin cancer; and decreased crop yields caused by environmental pollution and rising temperatures [11,12]. The negative impact on health caused by excessive carbon emissions is not only a serious problem for our society today, but also a serious constraint of sustainable development in the future [13]. Meanwhile, information and communication technologies (ICT) such as the Internet, big data, cloud computing, and artificial intelligence have been developed worldwide in recent years, and these new technologies have been used as key factors of production and combined with different economic activities to create a new economic form, i.e., the digital economy. With the development and widespread use of various digital technologies, the scale of the digital economy has also begun to grow rapidly in recent years. The 2019 China Academy of Information and Communications Technology (CAICT) calculation shows that the value added to the digital economy in 47 countries reached USD 31.8 trillion, an increase of USD 1.6 trillion from the previous year. Against the background of the growing trend of digitalization and the steady advancement of the global digital economy, the effective use of the digital economy to curb the growth of carbon emissions not only helps to alleviate environmental problems, but also plays an important role in the improvement of human health conditions. After summarizing previous studies, we found that existing studies have made preliminary explorations of the effects of the digital economy on carbon emissions, but have not yet reached a consensus. Firstly, in terms of ICT development, some studies argue that ICT development has contributed to the increase in energy consumption and carbon emissions [14]. Benefiting from the development of ICT technology, residents can participate in environmental monitoring more easily and make up for the government’s lack of environmental regulation [15]. Moreover, the development of the Internet can reduce energy consumption intensity [16] through various channels such as technological progress, financial development, and industrial structure upgrading and thus reduce carbon emissions. However, due to the rebound effect [17], the impact of ICT on environmental quality needs to be implemented simultaneously with corresponding policy measures to curb CO_2_ emissions. Some studies hold the opposite view, arguing that the large infrastructure demand for ICT construction leads to its implied carbon emissions far exceeding its direct carbon emissions reduction [18], leading to an increase in energy consumption and carbon emissions through rebound effects [19]. As part of the digital economy, digital finance has an impact on carbon emissions as well. It can dampen carbon emissions by facilitating economic growth, improving technological innovation [20], and other channels. Moreover, the development of the digital economy may also bring about the phenomenon of the digital divide [21,22], widen the income gap [23] and other effects.

In general, previous studies by scholars in this field have reached many important conclusions. However, there are limitations to previous studies. First, most of the previous studies only selected a certain aspect of the digital economy, such as ICT development and digital finance, as an entry point to discuss its relationship with carbon emissions. In addition, the theoretical analysis of the influence mechanism of the digital economy on carbon emissions has not been analyzed sufficiently. Furthermore, are the effects of the digital economy on carbon emissions exhibiting regional heterogeneity? Does the digital economy affect carbon emissions in neighboring regions through spatial spillover effects? The exploration of the above issues helps to clarify the intrinsic relationship of the digital economy and carbon emissions, which is critical for realizing sustainable development in China.

The main contributions of this research are as follows. Firstly, we construct a digital economy evaluation system from four perspectives: digital economy foundation, digital industrialization, industry digitization and digital economy penetration, which expands and improves the evaluation perspective of the digital economy. Secondly, the theoretical channel of the effect of digital economy on carbon emissions is proposed and verified with the help of the mediated-effects model, which improves the inherent logical relationship between the two. Third, a spatial econometric model is adopted to clarify the regional spillover effect of the digital economy on carbon emissions. This paper constitutes a reliable empirical reference for reducing carbon emissions worldwide, so as to alleviate the environmental problems and health problems brought by carbon emissions and achieve sustainable development. Since the carbon emissions of countries such as Brazil and India also continue to grow [24,25], the findings of this paper can provide a factual reference and theoretical basis for similar emerging economies to reduce their carbon emissions.

The remainder of the study is organized as follows. Section 2 conducts a literature review and theoretical analysis and proposes research hypotheses. Section 3 provides a description of the research methodology and data sources. Section 4 analyzes and discusses the empirical results. Section 5 provides conclusions and proposes relevant policy implications.

## 2. Literature Review and Research Hypotheses

### 2.1. Digital Economy and Carbon Emissions

The concept of the digital economy was first introduced by Tapscott [26] and has since been extensively researched and interpreted by scholars. Scholars generally agree that any economic output generated based on digital goods or services, whether they are wholly or partially dependent on digital technology, falls under the umbrella of the digital economy [27,28,29]. In addition to enabling the traditional economy, the booming digital economy has also enhanced the ability to integrate resources and innovation and has shown a significant contribution to reducing energy use intensity and carbon emissions [30,31,32]. First, from a macro perspective, the digital economy’s ability to integrate information and resources across regions can rebuild inter-regional economic structures, form positive feedback innovation mechanisms to coordinate the development of manufacturing and business, and thereby improve cross-regional synergistic innovation capabilities [33]. The thriving digital economy has enabled the penetration and integration of digital technologies into different parts of production to optimize production processes to reduce the wastage of resources and improve the efficiency of resource utilization [34] and total factor productivity [35]. The emergence of the digital economy is, to some extent, a reflection of the increase in technology and knowledge stock, and the development of digital processes has also effectively increased the ability to share information and knowledge [36]. The accumulation of knowledge is a key factor influencing innovation [37], so the digital economy can accelerate the innovation process to improve the efficiency of resource allocation and utilization and inhibit carbon emissions. The development of the digital economy has not only promoted technological innovation but also the emergence of new business models such as the “platform economy” and the “sharing economy”, which enable enterprises to achieve business development at relatively low costs [38]. Digital finance can solve the problem of financing constraints on the implementation of new business models by alleviating the mismatch of financial resources. New business models represent an increase in production and operational efficiency, and thus a reduction in carbon emissions. Secondly, from a meso perspective, the combination of the Internet with traditional industries has given industries more opportunities and possibilities, injecting new impetus into their development while also reducing carbon emissions. For example, the sharing economy brought about by the combination of the Internet and traditional industries, such as the sharing of trams and cars, has reduced the frequency of use of private cars by citizens, thus reducing energy consumption and carbon emissions [39]. With the information production factors provided by the digital economy, the ability of companies to access knowledge and information from outside is greatly enhanced, and the diffusion of information and technology between companies contributes to improving their innovation capabilities and performance [40,41]. In addition, the increasing complexity of economic activities has made it more difficult to regulate ecological and environmental issues, and the development of digital technology has provided the appropriate technical support for government environmental regulation. The use of big data, cloud computing, and remote sensing technology has provided the government with technical support for real-time dynamic monitoring of air quality, pollution emissions, and environmental carrying capacity [42,43]. Real-time monitoring of environmental problems and the formulation of relevant environmental policies for the government to curb regional carbon emissions provide the basis. Third, from a micro perspective, online offices and mobile payments have reduced the frequency of individual travel, paperless offices have reduced paper resource consumption, and increased digitization has greatly reduced transaction costs and resource consumption [44]. Various internet platforms act as digital media to both increases the public’s access to environmental perceptions and provide tools for the public to provide timely feedback on environmental information [15]. Digitalization penetrates all aspects of an individual’s life through various means, reducing carbon emission levels. According to the above theoretical analysis, Hypothesis 1 is proposed in this paper as follows.

**Hypothesis** **1.** *The development of the digital economy has effectively curbed the increase in carbon emissions.*

### 2.2. The Digital Economy, Mediating Mechanisms, and Carbon Emissions

Previous studies have shown that innovation and the upgrading of industrial structure play a key role in curbing carbon emissions [45,46,47]. Therefore, the mediating effect analysis in this paper also starts from the above two perspectives to explore the possible effects of the innovation effect and industrial structure upgrading effect on carbon emissions. The specific analysis is as follows.

#### 2.2.1. The Digital Economy, Innovation Mechanisms, and Carbon Emissions

In terms of the digital economy infrastructure, China’s vast territory requires a large amount of labor input to improve its digital economy infrastructure. In the process of building and improving infrastructure, the endogenous technological progress of industrial workers through the “learning by doing” effect [48] improves construction efficiency, which in turn reduces carbon emissions. Secondly, in terms of digital industrialization, the development of digital industrialization enhances the innovation capability and output of enterprises by enhancing the sharing of information and knowledge among enterprises to alleviate information asymmetry [40,41]. Similarly, in the digital age, the R&D departments of digital enterprises have to accelerate the innovation process to maintain their relative competitive advantage due to the full competition in the market [49], and the development and application of new technologies, such as recycling technologies and other green development technologies, can effectively reduce carbon emissions [50,51]. In terms of digitization of industries, the introduction of digital technologies such as AR and VR can enhance the intensity of interaction between industries and their target customers, thus increasing their product sales. The application of big data also provides companies with the necessary information for production decisions to reduce inventory accumulation and resource waste, thus reducing carbon emissions [52]. In addition, artificial intelligence, the Internet of Things, and cloud computing speed up the flow of new technologies among different enterprises, thus allowing them to integrate and develop with each other for the innovation of traditional industries [53]. Finally, in terms of digital economy penetration, digital finance, as a proxy variable of digital economy penetration, is innovative in its operation model compared to traditional financial institutions, avoiding the generation of carbon emissions from offline activities by transferring some financial activities from offline to online. With the help of massive information provided by big data technology, digital finance can reduce carbon emissions by rationally allocating and directing financial resources to environmentally-friendly sectors [54]. Since small and medium enterprises (SMEs) are the main drivers and subjects of innovation, and innovation projects are long-term and high-risk investment activities, financial constraints significantly constrain the R&D activities carried out by SMEs [4]. Digital finance evaluates SMEs at a low cost and provides these companies with financial resources that traditional financial institutions are not willing to provide to effectively alleviate financing constraints, which in turn increases corporate R&D investment and promotes the implementation and application of R&D results, thus promoting carbon emissions reduction. Through the above analysis, we propose the following hypothesis.

**Hypothesis** **2(a).** *The digital economy reduces carbon emissions by promoting innovation.*

#### 2.2.2. The Digital Economy, Industrial Structure Upgrading, and Carbon Emissions

The industrial structure upgrading makes the production factors transfer to industries with higher marginal returns and also makes the whole industrial structure greener [36,55], so the industrial structure upgrading also plays a crucial role in carbon emissions reduction [56,57]. From the perspective of the foundation of the digital economy, the massive construction of digital infrastructure improves the efficiency of corresponding industrial workers, and the efficiency improvement makes the surplus labor shift from the original industry to some service industries with a lower technological threshold. The shift of labor resources from manufacturing to the third industry promotes the upgrading of industrial structure, while the carbon emission level of the third industry is much lower than that of the secondary industry, thus suppressing the carbon emissions. From the perspective of digital industrialization, the relevant digital industries represented by big data, cloud computing, the Internet, the Internet of Things, etc., have higher technology content themselves, and the emergence of these low-carbon emission industries represents the adjustment and upgrading of industrial structure [30]. Secondly, digital industrialization provides a carrier for technology diffusion, which promotes inter-regional technology trade and spillover, which in turn play a key role in reducing carbon emissions [36,58,59,60]. From the perspective of industrial digitalization, the deep cross-fertilization of the digital economy and traditional industries accelerates the digital transformation process of traditional industries, and through the cross-regional integration and allocation of resources, the industrial structure can be adjusted to eliminate high-emission, high-pollution, and low-efficiency enterprises. Therefore, the promotion of industrial digitalization can also curb the growth of carbon emissions. Finally, in terms of digital economy penetration, the widespread application of digital technology can reduce frictional unemployment caused by information asymmetry. High-tech enterprises can obtain more factors of production to support their development by increasing marginal returns as they can obtain higher marginal returns. This positive feedback mechanism between factors of production and marginal returns further strengthens the development of high-tech enterprises. Digital finance can also guide the transformation and upgrading of industrial structure and thus reduce carbon emissions by directing the flow of financial resources to high-tech enterprises as well as green enterprises. Through the above analysis, we propose the following hypothesis.

**Hypothesis** **2(b).** *The digital economy reduces carbon emissions by promoting industrial structure upgrading.*

### 2.3. Spatial Spillover Effect of the Digital Economy and Carbon Emissions

Undoubtedly, the emergence of the digital economy has greatly weakened the inconvenience of economic interactions caused by geographical distance. Through ICT, the digital economy can enhance the efficiency of resource allocation and utilization in different regions [61] and effectively promote the flow of various production factors between regions. Moreover, due to the existence of externality, the conduct of an activity will not only have an impact on the local area, but also on the surrounding areas. The benefits that cannot be obtained locally due to the existence of such spatial externality are the spatial spillover effect [62,63]. Therefore, the existence of spatial spillover effects makes the digital economy have an impact not only on the carbon emissions in the region, but also on the carbon emissions in the surrounding areas. Previous studies have also used spatial econometric models to empirically analyze the influencing factors and spillover effects of carbon emissions [64,65]. Yilmaz [66] conducted an empirical analysis based on panel data from 1990 to 1997 in U.S. states to verify the spatial spillover effect of ICT technology, and later Liu [67] found that carbon emissions showed obvious spatial clustering characteristics by analyzing CO_2_ emissions at the city level in China. Xu [30] also conducted an empirical analysis by measuring the degree of ICT development, and the results showed not only that the development of ICT capital can improve the efficiency of carbon emissions in the region, but also that the spatial spillover effect on the surrounding areas cannot be ignored. Based on the theoretical analysis and empirical tests by previous scholars, Hypothesis 3 is proposed as follows.

**Hypothesis** **3.** *The digital economy affects the level of carbon emissions in neighboring regions through spatial spillover effects.*

**Figure 1:**
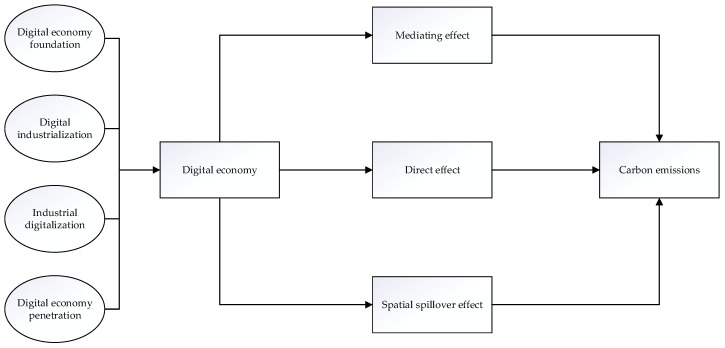
Mechanism analysis of the digital economy and carbon emissions.

After the mechanism analysis of the above hypotheses, we constructed a mechanism diagram of the role between the digital economy and carbon emissions, shown in Figure 1.

## 3. Research Methodology, Variable Selection, and Data Sources

### 3.1. Empirical Model Setting

With reference to previous scholars [65], we set up a fixed-effects regression model as Equation (1).

(1) $$Y_{it}=\beta_{0}+\beta_{1}Dig_{it}+\sum \beta_{k}X_{k}+\mu_{i}+\sigma_{t}+\varepsilon_{it}$$

where Y denotes the explained variable, the explanatory variable is the digital economy index (Dig); β represents the parameter to be evaluated; X denotes the control variables; i and t denotes region and year, respectively; and $\mu_{i}$ and $\sigma_{t}$ denote region and time fixed effects, respectively. $\varepsilon_{it}$ denotes the random error term.

Furthermore, in order to verify the mediating mechanism of the impact of the digital economy on carbon emissions as proposed above, we refer to the article by Wen [68] and set up a mediating effect model as follows.

(2) $$M_{it}=\alpha_{0}+\alpha_{1}Dig_{it}+\sum \alpha_{k}X_{k}+\mu_{i}+\sigma_{t}+\varepsilon_{it}$$

(3) $$Y_{it}=\gamma_{0}+\gamma_{1}Dig_{it}+\gamma_{2}M_{it}+\sum \gamma_{k}X_{k}+\mu_{i}+\sigma_{t}+\varepsilon_{it}$$

where M is the mediating variable; the rest of the variables are defined as in Equation (1). $\alpha_{1}\gamma_{2}$ is the coefficient of the mediating effect of the digital economy through mediating variables affecting the level of carbon emissions.

Since spatial data are spatially correlated [69], and CO_2_ emissions are a spatially dependent process, emissions from one province are influenced by carbon emissions from the rest of the neighboring areas while affecting the surrounding areas. Therefore, in order to verify the possible impact of CO_2_ emissions on neighboring areas, i.e., the spatial spillover effect proposed in Hypothesis 3, we also set the spatial econometric model as follows.

(4) $$Y_{it}=\alpha +\rho W_{ij}Y_{it}+\alpha_{1}Dig_{it}+\theta_{1}W_{ij}Dig_{it}+\sum \alpha_{k}X_{it}+\sum \theta_{k}W_{ij}X_{it}+\mu_{i}+\eta_{t}+\varepsilon_{it}$$

where ρ is the spatial autocorrelation coefficient, W denotes the weight matrix required for the spatial Durbin model, and the Queen matrix is used as the regression matrix. Since Hainan Province and the remaining provinces are not geographically adjacent, considering the reality of China, Hainan Province and Guangdong Province are set as neighbors to each other. θ denotes the coefficients of the spatial interaction terms of the explanatory and control variables. The rest of the variables are defined as above.

### 3.2. Description of Variables

#### 3.2.1. Explained and Mediating Variables

The explained variable selected for this study is the logarithm of carbon emissions (CE). According to the above hypotheses, we also selected two mediating variables: industrial structural upgrading (Struc), which is expressed as the share of the total output value of the secondary industry in GDP [70], and innovation level (Inv), which is expressed as the logarithm of the number of patents granted in each province.

#### 3.2.2. Explanatory Variable

The explanatory variable is the digital economy index (Dig), which is measured by the indicator system established in Table 1. Based on the interpretation and description of the connotation of the digital economy by previous scholars, we selected indicators from four aspects: the foundation of the digital economy, digital industrialization, industrial digitalization, and digital economy penetration. Among them, the digital economy foundation aspect mainly selects reference indicators from the level of digital infrastructure construction, including four indicators: Internet penetration rate, the number of cell phone base stations, the length of fiber optic cable lines per capita, and cell phone penetration rate. In terms of digital industrialization, indicators are mainly selected from the degree of development of the digital industry and the level of research and development, including the total amount of telecommunications business, fixed asset investment of information transmission and computer services, software industry, software business income, total technology contract turnover, the output value of the information service industry, R&D funding, and the number of patent applications granted. Industrial digitalization mainly focuses on the degree of digitalization of traditional industries in the digital era, and the number of websites per 100 enterprises, the proportion of enterprises with e-commerce transaction activities, and the e-commerce transaction amount are selected to represent them. Four indicators are selected for digital economy penetration: breadth of digital financial coverage, depth of digital financial usage, digital financial digitization, and online mobile payment level. The specific descriptions of each indicator are shown in Table 1.

The comprehensive evaluation methods mainly include two types of subjective evaluation methods and objective evaluation methods. The entropy method is one of the objective evaluation methods, which can make full use of the objective message included in the data to assign weights to each index. In order to avoid the impact caused by subjective factors, we measured the development level of the digital economy by the entropy method, and the specific steps are as follows.

1. Data dimensionless treatment.

Let $v_{ij}$ be the original data of the *j*-th indicator in the *i*-th evaluation object (*i* = 1, 2, 3, …, *n*; *j* = 1, 2, 3, …, *m*). In order to make the data of different calibers comparable and eliminate the difference of the dimension between the indicator data, the original data are standardized; if the indicator is a positive indicator, then the processing formula can be written as follows:

(5) $$x_{ij}=\frac{v_{ij}-min\left(v_{j}\right)}{max\left(v_{j}\right)-min\left(v_{j}\right)}$$

If the indicator is negative, the treatment formula can be written as follows:

(6) $$x_{ij}=\frac{max\left(v_{j}\right)-v_{ij}}{max\left(v_{j}\right)-min\left(v_{j}\right)}$$

$v_{ij}\left(i=1,2,3,\ldots ,n;\;j=1,2,3,\ldots ,m\right)$ is the raw data of the *j*-th index of the *i*-th evaluation object, and is the dimensionless value after standardization.

2. Calculate the entropy value and weight.

Let $y_{ij}$ be the weight of the *j*-th indicator in the *i*-th evaluation object, $e_{j}$ be the entropy value of the *j*-th indicator, and $g_{j}$ be the coefficient of variation in the *j*-th indicator, where the number of evaluation objects, is the weight of the first evaluation indicator (j=1,2,3,…,m). Each coefficient is calculated by the following formulas.

(7) $$y_{ij}=\frac{X_{ij}}{\sum_{i=1}^{n}X_{ij}}$$

(8) $$e_{j}=-\frac{1}{lnn}\sum_{i=1}^{n}y_{ij}ln\;y_{ij}$$

(9) $$g_{j}=1-e_{j}$$

(10) $$w_{j}=\frac{g_{j}}{\sum_{j=1}^{m}g_{j}}$$

3. Calculating the Digital Economy Score.

According to the weights of each indicator calculated by Equation (10) and the data of each indicator after standardization, the level of digital economy development can be calculated by Equation (11), where Dig represents the total digital economy score, and $w_{j}$ and $x_{ij}$ represent the weight and standardized value, respectively.

(11) $$Dig=\sum_{j=1}^{m}w_{j}x_{j}$$

#### 3.2.3. Control Variables

For the control variables, the following indicators were selected with reference to previous literature [30,71,72]. (1) The level of economic development, expressed as the logarithm of GDP per capita (PGDP). Previous studies have shown [14] that there is a significant correlation between the level of economic development and carbon emissions. (2) Urbanization (Urban), expressed as the ratio of urban population to total population. As a major source of carbon emissions, the large demand for energy consumption triggered by an increase in the urbanization rate may lead to an increase in carbon emissions [73] or reduce the level of carbon emissions through economies of scale. (3) Trade openness (Open), expressed as the ratio of total exports and imports to GDP [71]. In foreign trade, developed countries may transfer carbon emissions to countries in need of development by way of industrial transfer. (4) Foreign direct investment (FDI), expressed as the ratio of the total amount of foreign investment to GDP [74]. Foreign investment can increase carbon emissions by contributing to economic growth, and may also reduce carbon emission levels through technological factor inputs. (5) Total population (Pop), expressed as the logarithm of the total population in each province. An increase in population inevitably affects total carbon emissions [75]. (6) Environmental regulation (ER). According to previous studies, the degree of environmental regulation is directly related to carbon emissions. Moreover, the degree of greenery and the level of environmental regulation show a positive correlation—higher green areas are usually associated with higher levels of environmental regulation [76]. We selected green coverage as a proxy variable for environmental regulation.

### 3.3. Data Source

Due to the serious lack of data in Tibetan provinces, and the fact that data related to the digital economy were not published before 2009 and after 2019, only panel data from 30 provinces in mainland China from 2009 to 2019 were selected for regression analysis in this work. The data selected in this research are all from the statistical yearbooks of each province; the China Statistical Yearbook; the Institute of Digital Finance, Peking University; the CEADs database; and the CEE data platform. Some of the missing values are supplemented by interpolation. (See Table 2 for the descriptive statistics of the variables used in this study).

## 4. Empirical Results

### 4.1. Estimation Results of the Benchmark Model

Before conducting the benchmark regression, the Hausman test is performed to select the regression model, and the results of the Hausman test are shown in Table 3. The *p*-value of the Hausman test result is 0.0328, indicating the rejection of random effects. In addition to this, we also performed homoskedasticity and autocorrelation tests on the variables, and both *p*-values were zero, indicating that the model is BLUE. The regression results with stepwise addition of control variables are shown in Table 4. The regression results from the first column show that the development level of the digital economy and the level of carbon emissions have a significant negative relationship. After adding further control variables, although the coefficient of the digital economy changes from −0.762 to −0.484, the overall coefficient is still negative and significant at the 10% significance level, which reflects the robustness of the estimation results to some extent, thus verifying the Hypothesis 1 proposed in this research. Our empirical results further augment the findings of Li [72]. This also suggests that the Chinese government should vigorously play a positive role in curbing carbon emissions in the digital economy and fully releasing the dividends of the digital economy, thus making the development of the digital economy an effective means to cope with the climate and environmental problems caused by the increase in carbon emissions [72].

In terms of control variables, according to the results of the double fixed-effects model shown in column 6 of Table 4, the coefficient of the urbanization rate is 1.925 and significant at the 1% significance level. This means that a unit increase in urbanization level will result in a 1.925 unit increase in carbon emissions level, a result similar to previous scholars’ findings [77]. The reason is that the increase in urbanization rate needs to be supported by certain industrialization processes, and the increase in such industrialization processes will significantly increase the energy consumption and thus the carbon emission level. In addition, the increase in urbanization rate also means an increase in population density, and the basic demands of electricity, heating, and consumption caused by the population concentration will increase the carbon emissions level. The coefficient of the total population is 0.801 and significant at the 5% significance level, which implies that the increase in population size leads to significant growth in total carbon emissions. This result is also consistent with the findings of previous scholars [78], which indicate that the production and living activities resulting from population increase inevitably lead to increased energy demand and deterioration of environmental levels. Education and technology should be developed to fully utilize the population agglomeration effect to improve energy and environmental efficiency [36]. The coefficients of the remaining control variables are not significant after fixing the year and province, indicating that the remaining control variables are not significantly related to the explanatory variables from a statistical view.

### 4.2. Mediating Effect Model Regression Results

To further explore the mechanism of the mediating effect of the digital economy on carbon emissions, the mediating effect model set by Equations (2) and (3) is used to verify whether the digital economy affects carbon emissions through industrial structure upgrading and innovation. Most previous scholars who examine mediating effects adopt the stepwise test method proposed by Baron [79], but this method is not fully reliable for the test results of mediating effects, and it is easy to conclude insignificant results when the coefficients are actually significant. Later, scholars proposed the Bootstrap method [80] instead of the traditional Sobel method for mediating effects testing, which was widely used. Therefore, the Bootstrap method is also used in this work to verify the existence of mediating effects, and the results corresponding to the Bootstrap sampling 1000 times are reported. Columns 2 and 4 of Table 5 show the effect of industrial structure upgrading on carbon emission levels. The regression results show that the regression coefficient of the digital economy on industrial structure is −0.004, while the regression coefficient of industrial structure on carbon emissions is 1.019 and significant at 1% significance level. The main reason for this is that the proxy variable of industrial structure selected in this study is the share of secondary industry in GDP, which, combined with the Bootstrap test results in Table 6, proves that the development of digital economy curbs carbon emissions by promoting the upgrading of industrial structure. The development of the digital economy has effectively led to the emergence of new technologies and their integration with traditional industries, resulting in further optimization of the industrial structure. This result is consistent with our theoretical analysis above and the findings of previous studies [56]. Meanwhile, the Hypothesis 2(a) proposed above is confirmed. Columns 3 and 5 of Table 5 show the regression results of innovation effect on carbon emissions. As shown in Table 5, the regression coefficient of the digital economy effect on innovation is 2.460 and significant at the 1% level, which indicates that each unit of increase in the level of the digital economy will lead to a 2.460 unit increase in the level of innovation. The regression coefficient between the innovation effect and carbon emissions is −0.091 and passes the 5% significance test, which indicates that the increase in the innovation level can effectively reduce the total carbon emissions. Our empirical results further augment the findings of Baloch [54]. This, combined with the results of the Bootstrap test in Table 6, thus verifies our Hypothesis 2(b).

### 4.3. Analysis of Spatial Spillover Effects

**Figure 2:**
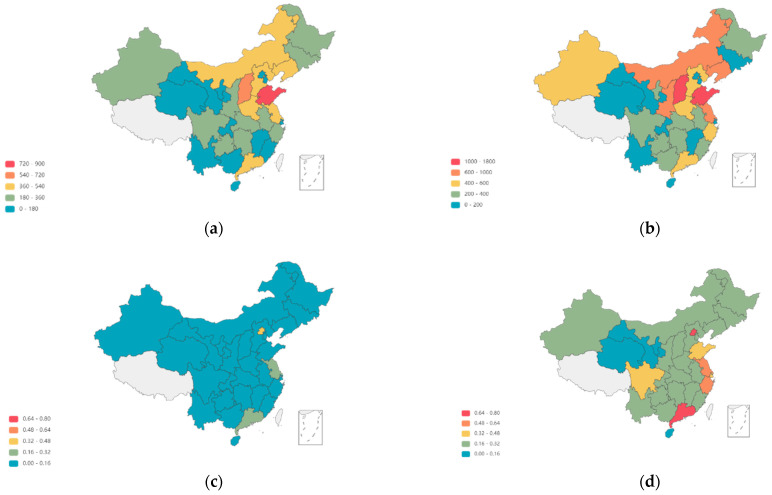
Carbon emissions and the development of the digital economy. (**a**,**b**) denote the level of carbon emissions in each province in 2009 and 2019, respectively; (**c**,**d**) denote the digital economy score in each province in 2009 and 2019, respectively.

Before conducting spatial econometric analysis, the existence of spatial correlation of the main variables should be tested. To have a clear understanding of the spatial distribution of the digital economy and carbon emissions, we plotted the digital economy and carbon emission levels of each province on a map, as shown in Figure 2. From Figure 2, it can be found that the carbon emissions show an obvious phenomenon of “high–high aggregation” and “low–low aggregation”, and most of the provinces have a significant increase in carbon emission levels in 2019 compared with the carbon emission levels in 2009. The development level of the digital economy was low in the early stage of the study period, except in Beijing. Later on, with the passage of time, the level of the digital economy in some central regions and the eastern coastal regions increased to different degrees and also shows regional correlation. In order to statistically verify whether there is a spatial correlation between these two variables, Moran’s I index is used in this paper to verify the spatial autocorrelation of the two variables for each year under the geographic adjacency matrix. The calculation formula is as in Equation (12), where $S^{2}=\frac{1}{n}\sum_{i=1}^{n}{\left(Y_{i}-\bar{Y}\right)}^{2}$, $\bar{Y}=\sum_{i=1}^{n}Y_{i}/n$, $Y_{i}$ is the carbon emissions in region *i*, and *W* is the spatial weight matrix, i.e., the spatial adjacency weight matrix. The Moran’s I index measures the spatial global autocorrelation, and its value range is [−1,1]. When the value range is (0,1], it means that there is a positive correlation, and the correlation degree increases with the value. There is no spatial correlation when the value is zero, there is a negative spatial correlation if the value range is [−1,0), and the correlation degree increases with the decreasing number.

(12) $$I=\frac{\sum_{i=1}^{n}\sum_{j=1}^{n}W_{ij}(Y_{i}-\bar{Y})(Y_{j}-\bar{Y})}{S^{2}\sum_{i=1}^{n}\sum_{j=1}^{n}W_{ij}}$$

From the Moran’s I index of carbon emission levels in Table 7, the *p*-value of carbon emissions is less than 0.05 and positive in all years, indicating that the carbon emissions of Chinese provinces show a significant positive spatial correlation. In contrast, the Moran’s I index of the digital economy index in Table 8 is positive, but not significant at the beginning and the end phase of the study. This is probably due to the fact that at the beginning of the study period, the prospect of digital economy development was still uncertain, and only some coastal provinces with high economic development levels had a good foundation for the digital economy development, while the rest of the regions had a low level of the digital economy, so the spatial correlation was not significant. At the end of the study phase, the benefits brought by the booming digital economy were valued by local governments, both infrastructure construction and market environment were improved compared to the early stage of the study phase, and the overall level of digital economy development was higher everywhere, which in turn led to an insignificant spatial correlation of the digital economy.

LM, LR, Robust LM, and Robust LR tests were performed on the set regression equations before the spatial Durbin model (SDM) regression; the results showed that the regression model could not be degraded to the spatial autoregressive model (SAR) or the spatial error model (SEM), and the Hausman test indicated that a fixed–effects model should be used, so the spatial Durbin model based on fixed effects was finally chosen for the regression analysis in this research. Table 9 shows the regression results of the SDM model, demonstrating that the digital economy shows a significant dampening effect on the total carbon emissions in local regions, which further verifies the Hypothesis 1. In addition, the coefficient of the spatial lag term of the digital economy, i.e., *WDig*, is 1.357 and is significant at the 1% level, indicating that the development of the digital economy not only affects carbon emissions in the local province but also in the neighboring provinces, and there is a spatial spillover effect. Furthermore, we draw on previous scholarly research and adopt a partial differential approach [81] to provide a detailed decomposition of the spatial spillover effect. As can be seen from column 3 of Table 9, the indirect effect of the digital economy on carbon emissions is positively significant at the 1% significance level, which means that there is a positive spillover effect of the development of the digital economy on neighboring regions. That is, the development of the digital economy will increase the carbon emissions of neighboring provinces. The empirical result is consistent with a previous study [67]. This is mainly due to the fact that the increase in the level of the digital economy in the province has forced the high-emission and high-polluting enterprises to move to neighboring provinces, and this cross-regional transfer of industries has increased the level of carbon emissions in these neighboring provinces. Through the above analysis, Hypothesis 3 is proved.

### 4.4. Analysis of Regional Heterogeneity

Due to the large number of provinces in China and the large regional differences in both the level of carbon emissions and the degree of the digital economy development, we further divided the 30 provinces in mainland China into three parts, East, Middle, and West, according to the regional division criteria of China (see Appendix A for provinces in each region), and regressed them separately in order to analyze their regional heterogeneity. The regression results in Table 10 show that the direct regression coefficients of the digital economy on carbon emissions in the three major regions are all negative, indicating that the increase in the level of the digital economy within each region has significantly reduced carbon emissions. In terms of the direct effect coefficient, the digital economy has the greatest inhibiting effect on carbon emissions in the central region with a regression coefficient of −13.148, followed by the western and eastern regions with coefficients of −4.542 and −1.120, respectively and all the coefficients passed the significance test. This is mainly due to the effective implementation of a series of strategies for the rise in the central region due to the policy support of the Chinese central government in recent years, which has led to a significant improvement in both the level of economic development and the state of infrastructure development in the central region. The development of digital technology and its deep integration with the traditional economy has unleashed the full potential of the digital economy, resulting in a significant increase in the level of digital economic development from an average value of 0.164 in 2009 to an average value of 0.224 in 2019, which has better curbed the increase in carbon emissions. Second, in terms of the coefficient of indirect effects, the western region has the largest spatial spillover effect, followed by the eastern region, while the central region has a negative but insignificant coefficient. This indicates that the market in the western region has more room for improvement due to the low level of development of the digital economy as a whole. The development of the digital economy in one province can not only effectively dovetail with the traditional industries in the region, but also lead to the improvement of the industrial level in neighboring provinces. Moreover, the development of the digital economy in a single province has created a significant demonstration effect, which has led to a reduction in carbon emissions in neighboring regions and therefore exhibits a significant spillover effect. In contrast, the spatial spillover effect in the eastern region is weaker than in the western region as the development of the digital economy in each province within the region is at a higher level, and the radiation capacity and driving effect of the development of the digital economy in each province on the surrounding provinces are more limited.

## 5. Robustness Test

To ensure the reliability of the empirical findings, we first replaced the explanatory variables with the Digital Financial Inclusion Index (DFI) measured by the Institute of Digital Finance of Peking University, which to some extent reflects the degree of digitization in China. As the starting year of the index is 2011, the regression data are reduced to 2011–2019 data for robustness testing. The first column of Table 11 shows the results of the regression with DFI as the explanatory variable, which shows that the coefficient of the explanatory variable to be −0.003, remaining negative and significant at the 5% level. Considering the possibility of a lag in the effect of digitization on carbon emissions, the regression in the Digital Finance Index with a first-order lag as the explanatory variable shows that the coefficient of the explanatory variable is still −0.003 and passes the significance test. In addition, the explained variable is replaced with the level of carbon emissions per capita (PCE) and then regressed, with the results shown in column 4 of Table 11, where the regression coefficient is −12.284 and significant at the 5% level, similar to the baseline regression results. This series of regression results also support the robustness of the above regression results.

## 6. Conclusions

With the deterioration of environmental conditions in recent years, the frequent occurrence of climate problems, and the growth of health problems accompanying the deterioration in environmental and climate problems, the transformation of development patterns and the reduction in pollution emissions play crucial roles in current and future sustainable development. The flourishing digital economy provides new impetus to transform development models, reduce carbon emissions, and develop sustainably. However, most studies have only considered a single dimension of the digital economy as an entry point to analyze the relationship between it and carbon emissions, such as ICT and carbon emissions, digital finance and carbon emissions, etc. This analysis cannot fully reflect the theoretical mechanism between the two. In view of this, we constructed an evaluation system for the degree of digital economy development from four perspectives. The mechanism of the effect between the two was analyzed in depth from several perspectives. Based on the panel data of 30 provinces in China from 2009 to 2019, the relationship between the digital economy and carbon emissions and the underlying mechanism was empirically tested in multiple dimensions using the fixed–effects model, the mediating effect model, and the SDM model. The findings show, firstly, that the digital economy significantly inhibits the growth of carbon emission levels and remains significant after gradually increasing the control variables, except for a change in the magnitude of the coefficient. The findings are still valid after a robustness test with replacement variables. Secondly, the effect of the digital economy in dampening carbon emissions shows regional heterogeneity, with the development of the digital economy in the central region having the greatest curbing effect on carbon emissions, followed by the western and eastern regions. Thirdly, the development of the digital economy effectively reduces carbon emissions through two mediating mechanisms: the innovation effect and the industrial structure upgrading effect. Fourthly, the inhibiting effect of the digital economy on carbon emissions shows a significant spatial spillover effect. The development of the digital economy has a dampening effect on carbon emissions both locally and in neighboring provinces. The spillover effect is greatest in the western region, followed by the eastern and central regions, showing regional heterogeneity.

## 7. Policy Implications

Based on the above findings, we propose the following policy implications.

First, strengthen the infrastructure of the digital economy to lay a solid foundation for the improvement of the digital economy. Increase the intensity of research and development to give continuous impetus to the development and transformation of digital technology. Promote the integration of traditional industries with digital technology vigorously to fully unleash the potential of digital technology. Enhance the application of digital technology to benefit more people and increase the penetration of the digital economy. Promote the level of the digital economy from multiple perspectives so that it can effectively curb the growth of carbon emissions.

Second, give full play to the innovation effect and industrial structure upgrading effect of the digital economy. Let innovation become the inexhaustible power to develop new industries and achieve industrial capacity expansion. Accelerate the process of the digital economy to promote the upgrading of industrial structure. Get rid of the traditional industrial development path dependence and thus achieve green low-carbon development.

Third, expand the channels of inter-regional dialogue and cooperation. The provinces should also strengthen cross-regional cooperation and assistance in the process of continuously promoting the deep integration of the digital economy and the traditional economy. Make full use of the advantages of the digital economy in the transmission of information and resource allocation, and promote the cross-regional flow of production factors in the digital economy so that spatial spillover effects can be effectively brought into play to achieve inter-regional synergy in carbon emissions reduction.

Finally, in addition to giving full play to the role of the digital economy in reducing carbon emissions, the Chinese government should explore more possible mechanisms to effectively address the growth of carbon emissions—for example, by accelerating the research and development and application of renewable energy and clean energy to break away from the energy path dependence of the traditional development approach, and by accelerating the promotion of carbon emissions trading to effectively achieve carbon emissions reduction through market mechanisms.

The limitations of the study are as follows. First, due to the limitations of data and space, we can only analyze the relationship between the digital economy and carbon emissions in the Chinese policy environment. Future research could explore whether the digital economy in developed countries has a different impact on carbon emissions than in China. Second, more impact mechanisms on carbon emissions should be ascertained to better achieve carbon reduction and sustainable development.

## Figures and Tables

**Table 1:** Evaluation system of the digital economy.

Target Level	Criterion Level	Index Level	Unit	Indicator Direction
Digital economy	Digital economy foundation	Internet penetration rate	%	+
		Number of cell phone base stations	Million	+
		Length of fiber optic cable lines per capita	Km/million people	+
		Cell phone penetration rate	%	+
	Digital industrialization	Total amount of telecommunications business	Billion yuan	+
		Fixed asset investment of information transmission and computer services, and software industry	Billion yuan	+
		Software business income	Million yuan	+
		Total technology contract turnover	Million yuan	+
		Number of patent applications granted	/	+
		R&D funding	Billion yuan	+
		Output value of information service industry	Billion yuan	+
	Industrial digitalization	The number of websites per 100 enterprises	/	+
		Proportion of enterprises with e-commerce transaction	%	+
		E-commerce transaction amount	Million yuan	+
	Digital economy penetration	Breadth of digital financial coverage	/	+
		Depth of digital financial usage	/	+
		Digital financial digitization	/	+
		Online mobile payment level	/	+

**Table 2:** Statistical description of the variables.

Variables	Observations	Mean	Std. Dev.	Minimum	Maximum
CE	330	5.6050	0.7730	3.5680	7.4380
Dig	330	0.1450	0.1130	0.0221	0.6870
Struc	330	0.4440	0.0867	0.1620	0.5900
Inv	330	9.8140	1.4900	5.5760	13.1800
PGDP	330	10.3100	0.4200	9.2170	11.2800
Urban	330	0.5640	0.1270	0.2990	0.8960
Open	330	0.2710	0.3110	0.0127	1.5480
FDI	330	0.0207	0.0158	0.0001	0.0819
Pop	330	8.1970	0.7380	6.3240	9.3520
ER	330	39.0200	3.9300	27.1000	55.1000

**Table 3:** Hausman test results.

Variables	RE	FE	Difference	S.E.
Dig	−0.616	−0.738	0.122	0.028
FDI	−0.376	−1.004	0.628	0.183
PGDP	0.177	0.164	0.012	0.000
Urban	1.848	1.996	−0.148	0.139
Open	0.072	−0.090	0.162	−0.048
POP	1.121	0.794	0.327	0.351
ER	0.011	0.012	−0.001	0.000
chi2(7) = 15.26		Probability > chi2 = 0.0328		

**Table 4:** Benchmark regression results.

Variables	FE1	FE2	FE3	FE4
Dig	−0.762 ***	−0.813 ***	−0.568 **	−0.484 *
	(−3.244)	(−3.371)	(−2.049)	(−1.747)
FDI		0.571	0.161	0.440
		(0.662)	(0.187)	(0.504)
PGDP		0.067	−0.084	−0.091
		(0.911)	(−0.967)	(−1.046)
Urban			2.000 ***	1.925 ***
			(3.004)	(2.888)
Open			−0.056	−0.008
			(−0.408)	(−0.057)
Pop				0.801 **
				(2.126)
ER				0.007
				(1.249)
Constant	5.417 ***	4.735 ***	5.257 ***	−1.449
	(189.316)	(6.490)	(7.103)	(−0.464)
Year FE	YES	YES	YES	YES
Province FE	YES	YES	YES	YES
Observations	30	30	30	30
R-squared	0.407	0.410	0.435	0.447

Note: *t*-statistics in parentheses; *** *p* < 0.01, ** *p* < 0.05, * *p* < 0.1.

**Table 5:** Mediating effect regression results.

Variables	Y = Struc	Y = Inv	Y = CE	Y = CE
Dig	−0.004	2.460 ***	−0.480 *	−0.262
	(−0.098)	(5.757)	(−1.751)	(−0.900)
Struc			1.019 ***	
			(2.743)	
Inv				−0.091 **
				(−2.368)
FDI	0.070	−0.927	0.369	0.356
	(0.503)	(−0.689)	(0.427)	(0.411)
PGDP	0.137 ***	0.127	−0.230 **	−0.079
	(9.964)	(0.951)	(−2.308)	(−0.919)
Urban	0.062	0.133	1.862 ***	1.937 ***
	(0.588)	(0.129)	(2.823)	(2.929)
Open	−0.042 *	0.209	0.035	0.011
	(−1.927)	(0.982)	(0.254)	(0.080)
Pop	0.053	3.797 ***	0.746 **	1.145 ***
	(0.893)	(6.542)	(2.002)	(2.856)
ER	0.000	0.013	0.007	0.008
	(0.062)	(1.544)	(1.253)	(1.470)
Constant	−1.357 ***	−31.640 ***	−0.066	−4.315
	(−2.750)	(−6.582)	(−0.021)	(−1.299)
Year FE	Yes	Yes	Yes	Yes
Province FE	Yes	Yes	Yes	Yes
Observations	330	330	330	330
R-squared	0.863	0.547	0.462	0.458

Note: *t*-statistics in parentheses; *** *p* < 0.01, ** *p* < 0.05, * *p* < 0.1.

**Table 6:** Bootstrap test results.

Variables		Coefficient	*z*-Value	*p*-Value
Struc	Direct effect	−1.378	−3.56	0.000
	Indirect effect	−0.691	−3.01	0.003
Inv	Direct effect	−1.598	−4.26	0.000
	Indirect effect	−0.472	−2.95	0.003

**Table 7:** Moran’s I of carbon emissions.

Year	Moran’s I	E	sd	*z*	*p*
2009	0.183	−0.034	0.012	1.964	0.025
2010	0.192	−0.034	0.012	2.048	0.020
2011	0.208	−0.034	0.012	2.187	0.014
2012	0.201	−0.034	0.012	2.125	0.017
2013	0.227	−0.034	0.012	2.362	0.009
2014	0.213	−0.034	0.012	2.236	0.013
2015	0.215	−0.034	0.012	2.254	0.012
2016	0.199	−0.034	0.012	2.108	0.018
2017	0.191	−0.034	0.012	2.034	0.021
2018	0.186	−0.034	0.012	1.992	0.023
2019	0.176	−0.034	0.012	1.903	0.029

**Table 8:** Moran’s I of the digital economy.

Year	Moran’s I	E	sd	*z*	*p*
2009	0.099	−0.034	0.012	1.209	0.113
2010	0.114	−0.034	0.012	1.340	0.090
2011	0.086	−0.034	0.012	1.090	0.138
2012	0.146	−0.034	0.012	1.629	0.052
2013	0.130	−0.034	0.012	1.482	0.069
2014	0.132	−0.034	0.012	1.503	0.066
2015	0.134	−0.034	0.012	1.525	0.064
2016	0.119	−0.034	0.012	1.388	0.083
2017	0.104	−0.034	0.012	1.247	0.106
2018	0.100	−0.034	0.012	1.218	0.112
2019	0.094	−0.034	0.012	1.161	0.123

**Table 9:** Spatial Durbin model regression results.

Variables	Main	Wx	LR_Direct	LR_Indirect	LR_Total
Dig	−0.854 ***	1.357 ***	−0.865 ***	1.335 ***	0.470
	(−3.382)	(3.043)	(−3.377)	(3.111)	(0.995)
FDI	0.556	6.260 ***	0.438	6.091 ***	6.530 ***
	(0.701)	(3.153)	(0.581)	(3.322)	(3.181)
PGDP	−0.032	−0.198	−0.022	−0.182	−0.204
	(−0.422)	(−1.448)	(−0.292)	(−1.423)	(−1.429)
Urban	1.679 ***	2.724 **	1.606 ***	2.444 **	4.050 ***
	(2.808)	(2.313)	(2.856)	(2.239)	(3.419)
Open	−0.094	−0.056	−0.087	−0.030	−0.117
	(−0.778)	(−0.215)	(−0.705)	(−0.115)	(−0.421)
Pop	0.854 **	1.198	0.854 ***	1.084	1.938 **
	(2.529)	(1.619)	(2.599)	(1.502)	(2.239)
ER	0.008 *	0.001	0.008	0.000	0.008
	(1.695)	(0.052)	(1.634)	(0.012)	(0.701)
Year FE	Yes	Yes	Yes	Yes	Yes
Province FE	Yes	Yes	Yes	Yes	Yes
Observations	330	330	330	330	330
R-squared	0.413	0.413	0.413	0.413	0.413

Note: *z*-statistics in parentheses; *** *p* < 0.01, ** *p* < 0.05, * *p* < 0.1.

**Table 10:** Spatial Durbin model regression results by region.

Variables	East	Middle	West
Dig	−1.199 ***	−13.057 ***	−5.197 **
	(-5.092)	(−4.549)	(−2.484)
Wx	−1.035 **	1.219	−17.991 ***
	(-2.484)	(0.354)	(−2.975)
LR_Direct	−1.120 ***	−13.148 ***	−4.542 **
	(-4.716)	(−4.189)	(−2.239)
LR_Indirect	−0.746 **	−1.411	−15.748 ***
	(-2.034)	(−0.323)	(−2.948)
LR_Total	−1.866 ***	−14.559 **	−20.289 ***
	(-4.193)	(−2.217)	(−3.225)
ρ	−0.204 **	0.190 **	−0.132
	(−2.201)	(2.222)	(−0.853)
Control Variables	Yes	Yes	Yes
Year FE	Yes	Yes	Yes
Observations	121	88	121
R-squared	0.965	0.055	0.011

Note: *z*-statistics in parentheses; *** *p* < 0.01, ** *p* < 0.05.

**Table 11:** Robustness checks.

Variables	Y = CE	Y = CE	Y = PCE
	X = DFI	X = L.DFI	X = Dig
X	−0.003 **	−0.003 **	−12.284 **
	(0.001)	(0.001)	(5.334)
FDI	0.986	0.534	22.180
	(0.883)	(0.867)	(16.804)
PGDP	−0.017	−0.001	−5.605 ***
	(0.097)	(0.087)	(1.670)
Urban	0.769	0.124	38.790 ***
	(0.802)	(0.776)	(12.822)
Open	0.185	0.230 *	−3.716
	(0.134)	(0.136)	(2.660)
Pop	1.905 ***	2.411 ***	6.444
	(0.525)	(0.582)	(7.243)
ER	−0.009	−0.009	0.126
	(0.006)	(0.006)	(0.104)
Constant	−9.847 **	−13.750 ***	−11.887
	(4.167)	(4.563)	(59.991)
Year FE	Yes	Yes	Yes
Province FE	Yes	Yes	Yes
Observations	270	240	330
R-squared	0.175	0.187	0.250

Note: “L.” in the table denotes the first-order lag of the variables; *t*-statistics in parentheses; *** *p* < 0.01, ** *p* < 0.05, * *p* < 0.1.

## Data Availability

The data involved in this study are all from public data.

## Appendix A

The eastern region includes Beijing, Tianjin, Hebei, Shanghai, Liaoning, Jiangsu, Zhejiang, Fujian, Shandong, Guangdong, Hainan; the central region includes Shanxi, Jilin, Heilongjiang, Anhui, Jiangxi, Henan, Hubei, Hunan; the western region includes Inner Mongolia, Guangxi, Chongqing, Sichuan, Guizhou, Yunnan, Shaanxi, Gansu, Qinghai, Ningxia, Xinjiang.
